# Adult Frass Provides a Pheromone Signature for *Drosophila* Feeding and Aggregation

**DOI:** 10.1007/s10886-016-0737-4

**Published:** 2016-08-18

**Authors:** Ian W. Keesey, Sarah Koerte, Tom Retzke, Alexander Haverkamp, Bill S. Hansson, Markus Knaden

**Affiliations:** Department of Evolutionary Neuroethology, Max Planck Institute for Chemical Ecology, Jena, Germany

**Keywords:** Olfactory, Gustatory, Chemical ecology, *Drosophila*, Frass, Feces, Pheromones, Insect behavior

## Abstract

Adult *Drosophila melanogaster* locate food resources by using distinct olfactory cues that often are associated with the fermentation of fruit. However, in addition to being an odorous food source and providing a possible site for oviposition, fermenting fruit also provides a physical substrate upon which flies can attract and court a potential mate. In this study, we demonstrate that *Drosophila* adults are able to recruit additional flies to a food source by covering the exposed surface area with fecal spots, and that this recruitment is mediated via olfactory receptors (Ors). Analyses of the deposited frass material demonstrates that frass contains several previously studied pheromone components, such as methyl laurate (ML), methyl myristate (MM), methyl palmitate (MP), and 11-*cis*-vaccenyl acetate (cVA), in addition to several cuticular hydrocarbons (CHCs) that are known to be behaviorally active. Moreover, this study also demonstrates that adult feeding is increased in the presence of frass, although it appears that Ors are less likely to mediate this phenomenon. In summary, the frass deposited by the fly onto the fruit provides both pheromone and CHC cues that lead to increased feeding and aggregation in *Drosophila*. This research is the first step in examining *Drosophila* frass as an important chemical signature that provides information about both the sex and the species of the fly that generated the fecal spots.

**Electronic supplementary material:**

The online version of this article (doi:10.1007/s10886-016-0737-4) contains supplementary material, which is available to authorized users.

## Introduction

The pheromone system of *Drosophila* has been extensively studied, and previous research provides detailed information on the chemical identity of behaviorally relevant compounds that are generated by male and female flies (Auer and Benton 2016). This broad area of research also delves deeply into the neuronal mechanisms for both the detection and the decision-making of the fly in response to the presence of these pheromones, including the governance of complex multi-modal phenomena such as mate recognition and courtship. Recently, several important olfactory receptor ligands were uncovered, including methyl laurate (ML), methyl myristate (MM), and methyl palmitate (MP), which are some of the best known ligands for pheromone receptors Or47b and Or88a (Dweck et al. 2015). In addition, work by Lin et al. (2016) also suggests that myristic acid, palmitoleic acid, and palmitic acid could also act as important ligands as well. These two new studies provide olfactory ligands that fit nicely into the already established model for the neuronal activation of these circuits; however, the origin and production site of these fatty acid derived ligands has not yet been determined.

Feces collected from various insects has been previously studied for several attributes such as chemistry, shape, and color (Kuhns et al. 2012; Shao et al. 2012; Tumlinson et al. 1969; Wayland et al. 2014). In the case of the boll weevil, the examination of frass provided the behavioral relevance and eventually the identification of specific pheromone components that were otherwise difficult to isolate from adult odor collections or from the associated chemical analyses of courtship (Tumlinson et al. 1969). More recently the importance of fecal pheromones in aggregation behavior also was demonstrated in the German cockroach, *Blattella germanica*, where researchers showed that this insect emits highly attractive carboxylic acids in healthy adult feces (Wada-Katsumata et al. 2015). It also has been noted that frass can provide behaviorally relevant cues to parasitoids, such as wasps that target larvae of the diamondback moth (Reddy et al. 2002). Thus, frass across the order Insecta already has been established as a well-known substrate for behaviorally relevant odor cues.

Previous examination of *Drosophila melanogaster* frass has yielded information concerning the physical properties such as shape, size, and optical density of fecal droplets. These studies provided interesting differences in frass that depend on mating status and sex of each *D. melanogaster* fly that was tested (Wayland et al. 2014). In addition, researchers also have examined frass in regard to the quantification of fecal production, as well as the concentration of fecal material, in order to generate data on total excretion and water reabsorption (Linford et al. 2015; Urquhat-Cronish and Sokolowski 2014; Wayland et al. 2014). These studies showed the importance of frass in non-invasive studies of *Drosophila* metabolism and suggested that frass could be used as a metric for assessing general health, especially as it pertains to either nutrient or microbial stress. However, no previous studies have examined *Drosophila* frass in regard to its chemical properties or tested this digestive byproduct for any behavioral relevance. Here, we first document strong attraction of *Drosophila* adults towards frass, as well as demonstrate the presence of several CHCs and pheromones. We also provide a protocol for the collection of fecal material, as well as potential procedures for the examination of sex- and species-specific differences between fecal collections across this genus of flies.

## Materials and Methods

### Fly Stocks

All wildtype fly lines, including *D. simulans* (14,021–0251.195), *D.erecta* (14,021–0224.01), *D. mauritiana* (14,021–0224.01), *D. virilis* (15,010–1051.00), *D. suzukii* (14,023–0311.01), *D. biarmipes* (14,023–0361.10), and *D. pseudoobscura* (14,021–0121.94) were obtained from the UCSD Drosophila Stock Center (www.stockcenter.ucsd.edu). All experiments with wild-type *D. melanogaster* were carried out with Canton-S (WTcs, stock #1), which were obtained from the Bloomington *Drosophila* Stock Center (www.flystocks.bio.indiana.edu). Stocks were maintained according to previous studies, and for all behavioral experiments we used 2–5 d-old flies of both sexes.

### Stimuli and Chemical Analysis

All of the synthetic odorants that were tested and confirmed were acquired from commercial sources (Sigma, www.sigmaaldrich.com and Bedoukian, www.bedoukian.com) and were of the highest purity available. Stimuli preparation and delivery for behavioral experiments followed previously established procedures, and any headspace collection of volatile odors was carried out according to standard procedures (Keesey et al. 2015). Blueberries were selectively used for fruit experiments since *D. melanogaster* could not penetrate or oviposit through the hardened surface of the berries. In addition, the small size of the blueberry allowed the use of intact, completely sealed fruit, which further prevented *D. melanogaster* from gaining any access beneath the surface or skin of the berry. GC-MS analyses were performed on all volatile and insect body wash collections as described previously (Dweck et al. 2015). The NIST mass-spectral library identifications were confirmed with chemical standards where available.

### Frass Collections

The sides of rearing vials that contained 100 adult flies were scraped after 1 wk. with a flat, rounded-end micro spatula. Each rearing vial could be separated into distinct zones of pupation as well as frass deposition (Supplemental 3), and thus no larvae or pupal cases were included in these frass collections. After scraping was completed, 150–200 mg of frass were added to either 1 ml of water, methanol, or hexane solvent. After 24 h, collected material was filtered through sterilized paper disks to remove large particles, and then these frass infused solvents were used in behavioral trials with the addition of mineral oil.

### Behavioral Assays

Trap assays were performed with 2–5 d-old flies as previously described (Keesey et al. 2015; Knaden et al. 2012), but with an additional 200 μl of light mineral oil (Sigma-Aldrich, 330,779–1 L) that was added to capture and drown flies upon contact with the treatment or control within the container. All behavioral traps consisted of 60 ml plastic containers (Rotilabo sterile screw cap, Carl Roth GmbH, EA77.1), with one trap used as a solvent control and the other containing the treatment (Fig. 3f). All trap experiments were repeated using water, methanol, or hexane as solvents for the frass collections. While all solvents generated significant attraction towards frass when compared to the control, water was the best solvent for behavior, but it could not be used for further GC-MS analyses, thus methanol was utilized instead for all additional experiments with *Drosophila* frass, as it had the closest polarity to water. Flywalk trials also were conducted as described previously (Steck et al. 2012; Thoma et al. 2014; Supplemental Fig. 5). In short, 15 flies were placed individually into parallel glass tubes. During the experiment, flies were exposed continuously to a humidified airflow of 20 cm/s (70 % relative humidity, 20 °C). Flies were presented repeatedly with air pulses from the head space of frass solved in water, or to pulses of water alone, at an interstimulus interval of 90 s for 8 h. The 500 ms pulsed air stimuli were added to the continuous airstream and thus traveled through the glass tubes at a constant speed. The individual flies’ movements before and after stimulus arrival were monitored under red-light conditions using advanced video-tracking software (Steck et al. 2012; Thoma et al. 2014).

### Feeding Assays

All tested flies were 2–5-d-old, included both males and females, and were starved beforehand for 18–20 h with constant access to water. Flies then were cooled for 5 min at -20 °C to assist in their transfer to the petri dish arena. Basic feeding solutions consisted of water with 5 % sucrose and 5 % baker’s yeast, and experiments were conducted with or without colored dye markers (red and blue). Frass was added to treatment solutions, and included 150–200 mg of material per 1 ml of sugar water. After the 20 flies entered the arena, observations of fly feeding behavior were made at 2 min intervals for 30 min. Flies that fed on dye markers then were frozen at -20 °C, and images were taken for counting and additional analyses. The capillary feeder (CAFÉ) assays utilized glass micropipettes with liquid media that were filled by capillary action, and then inserted through pipette tips into the container holding the adult flies (modified from Ja et al. 2007). One capillary contained the control (5 % sucrose), while the other contained the treatment (5 % sucrose plus frass), and the volume consumed from each side was measured after a set duration of fly feeding.

## Results

### Fecal Deposits on Fruit

**Fig. 1:**
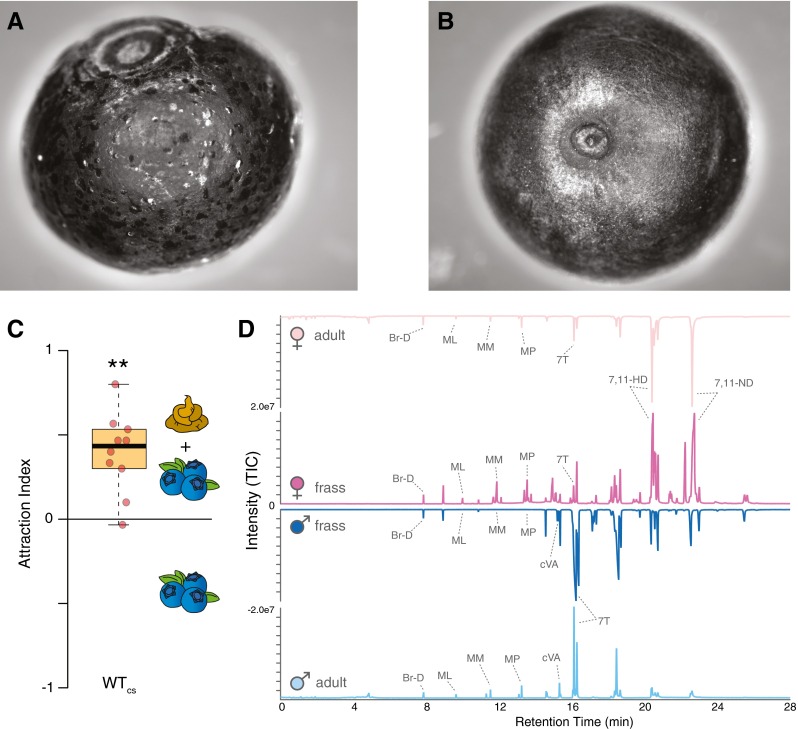
**a** Image of a blueberry that was exposed to *Drosophila melanogaster* flies for 24 h, where the flies randomly distribute droplets of feces to cover the entire exposed surface area of the fruit. **b** Blueberry without exposure to flies. **c** Trap assays using fruit with and without previous fly contact (i.e., with and without fecal spots), where the fruit with *Drosophila* frass was preferred over the fruit alone. Attraction indices were calculated as (O-C)/T, where O is the number of flies observed in the treatment trap, C is the number of flies in the control trap, and T is the total number of flies used in the trial. **d** Adult male and female chemical profiles were established via short body washes in solvent, and the same procedures were used for GC-MS analyses of frass. Both male and female frass contained significant amounts of previously identified pheromone components, and each frass sample most closely resembles the sex of the adult that produced it. (Br-D, bromodecane [internal standard]; ML, methyl laurate; MM, methyl myristate; MP, methyl palmitate; 7 T, (Z)-7-tricosene; cVA, cis-vaccenyl acetate; 7,11-HD, (7Z, 11Z)-heptacosadiene; 7,11-ND, (7Z, 11Z)-nonacosadiene)

*Drosophila* adults that had access to fruits, deposited fecal spots directly onto the fruit surface area using randomly spaced, often non-overlapping droplets (Fig. 1a, b). Surface washings of the fruit with and without deposited fecal spots, and solvent extractions of frass material alone revealed that several behaviorally important compounds were present in association with these fecal droplets, including the recently described pheromone components methyl laurate (ML), methyl myristate (MM), and methyl palmitate (MP), as well as their corresponding acids (lauric acid, myristic acid, palmitoleic acid, and palmitic acid). In a trap assay, when *Drosophila* adults were allowed to choose between the odor of fruit alone, and the odor of fruit that had been in contact with other *Drosophila*, the majority of flies selected the fruit with previous exposure to conspecifics (Fig. 1c). To ascertain the chemical profile of the frass alone, the fecal deposits were collected along the sides of the clear plastic rearing vials and placed into three solvents, which included water, methanol and hexane (Fig. 1d; Supplemental Fig. 3). Although water and methanol extracts were the most consistently attractive, all three fecal solvent extractions produced attraction in WT flies (i.e., wildtype flies of the Canton S strain) and w1118 control flies (i.e., white eye flies that carry the same genetic background as the other tested mutant fly lines). It also was noted that water completely dissolved the fecal material while hexane did not, suggesting that the frass contains predominantly polar compounds.

### Differences between Male and Female Frass

To test for any differences between male and female frass, newly emerged virgin flies were collected and placed into separate rearing vials based on sex. Subsequent fecal collection was completed as described previously (Supplemental Fig. 3), and this sex-specific frass material was added to methanol for further chemical analyses. By comparing adult body washes to these sex-specific fecal profiles by using GC-MS, it was demonstrated that frass contains information regarding the sex of the fly (Fig. 1d; Supplemental 6 A, B), and moreover, that the chemical signature of the frass matches most closely the *Drosophila* adult that produced it (Fig. 1d). More specifically, the GC-MS data showed that feces of both sexes contain the recently described pheromones ML, MM, and MP, while male feces contains a large amount of 11-*cis*-vaccenyl acetate (cVA) and 7-tricosene (7 T), and that female feces contains higher amounts of (7Z-11Z)-heptacosadiene (7,11-HD) and (7Z,11Z)-nonacosadiene (7,11-ND), which matches previously reported adult pheromone and adult CHC profile differences between the two sexes (Auer and Benton 2016; Dweck et al. 2015).

### Attraction Towards Frass

**Fig. 2:**
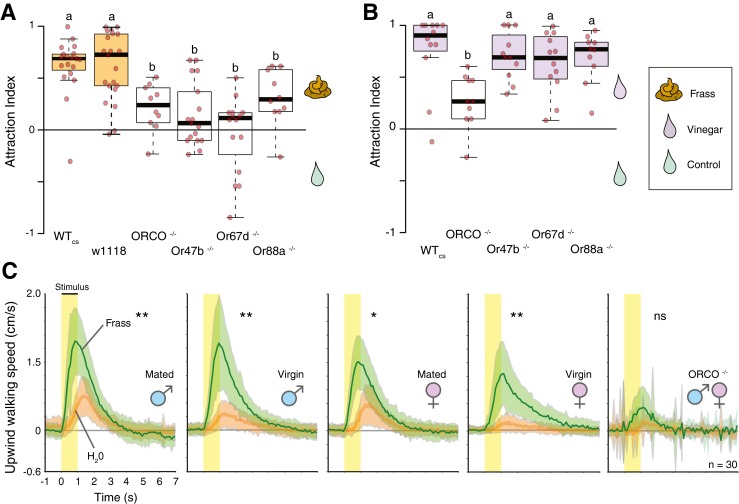
Attraction indices from trap assays containing either **a** frass or **b** vinegar. Data includes flies deficient in either Orco or pheromone-specific Ors, and also shown are the corresponding responses of wild type (WTcs, *Drosophila melanogaster* Canton S) and other transgenic control flies (w1118, white eye). Attraction indices were calculated as (O-C)/T, where O is the number of flies observed in the treatment trap, C is the number of flies in the control trap, and T is the total number of flies used in the trial. **c** Responses to frass vs. the water control in the Flywalk, which includes behavioral response data from mated and virgin, as well as male and female adults. Both males and females are significantly attracted towards frass at all time intervals (*P* < 0.01). Males were significantly more attracted than females, regardless of mating status (*P* < 0.01). Tests with Orco flies did not produce any significant attraction towards frass

**Fig. 3:**
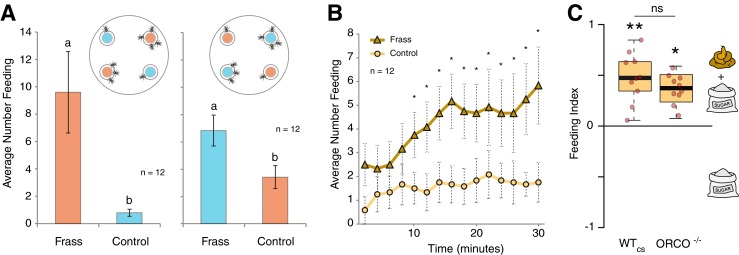
Assays comparing feeding on sugar solution alone vs. sugar solution that also contain frass. Trials were conducted with both red and blue dye. Feeding behavior of each fly was documented based on the amount of red or blue dye in the abdomen after a 30 min exposure to the food (see also Supplementary Fig. 4). **b** Numbers of flies that were observed feeding at the frass-containing and the control food sources during 2 min intervals of direct observation for a total of 30 min. Flies contacted and fed upon frass-containing sugar solutions significantly more than the controls. **c** Feeding indices of wildtype and Orco mutant flies using a CAFÉ assay with 5 % sucrose solution either with or without frass. Significant differences are denoted by letters or asterisks (ANOVA followed by Tukey’s test; *P* < 0.05). Error bars represent SEM

To test the behavioral relevance of frass, trap assays were used to compare the solvent control against the fecal collections. For water, methanol and hexane solvents, the frass was significantly more attractive than the evaporated solvent controls (Fig. 2a; WT, Canton S and w1118, white eyes; methanol data shown). Next, to examine the importance of odorant receptors, mutant flies lacking a functional olfactory co-receptor (Orco) were tested for their attraction towards frass. These mutant flies displayed a significantly reduced but still significant behavioral preference for frass, suggesting that at least part of the attraction towards frass was mediated by olfactory sensory neurons expressing odorant receptors, but also that other types of receptors were involved. To further address the importance of previously identified pheromone components in the attraction towards frass, multiple mutant fly lines were utilized that were only deficient in specific pheromone receptors, including Or47b (detecting ML), Or67d (detecting cVA), and Or88a (detecting ML, MM, and MP). All three of these mutant fly lines demonstrated reduced attraction towards frass, and all three were significantly different from the two control fly lines (WTcs and w1118); moreover, these mutant fly lines were not statistically different from the ORCO mutant line, further suggesting the important role of olfactory pheromone receptors in the behavioral attraction of adult flies towards frass material (Fig. 2a). To test that all mutant lines (Or47b, Or67d, Or88a) were still behaviorally functional, additional trap assays were conducted with vinegar, which is a general attractant that does not rely on pheromone receptors for attraction (Fig. 2b). While Orco mutant flies were still deficient in their attraction towards vinegar, the three pheromone receptor mutants (Or47b, Or67d, Or88a) all displayed the same level of attraction to vinegar as both control lines, suggesting that these mutant flies exhibited normal behavior towards attractants that do not rely on pheromone detection. Therefore we conclude that the reduced response to frass by these three pheromone mutant lines is due to their loss of specific pheromone Ors. To further test the role of frass in aggregation and attraction, the Flywalk was utilized as well (Thoma et al. 2014; Supplemental Fig. 5D). Using this behavioral paradigm it was demonstrated that the odor of frass was indeed more attractive than the water control for both virgin and mated males (*P* < 0.01), as well as for both virgin and mated females (*P* < 0.01) (Fig. 2c), with flies reaching walking speeds towards frass odor that exceeded those previously published with some of the best *Drosophila* attractants such as ethyl acetate and ethyl butyrate (Thoma et al. 2014). There was no significant difference between mated and virgin males (*P* > 0.05), nor was there any significant difference between mated and virgin females (*P* > 0.05). However, mated males were significantly more attracted than mated females towards frass (*P* < 0.01), and virgin males were more attracted than virgin females (*P* < 0.01). As was shown with the previously reported trap assays, the Orco mutant line again was significantly less attracted to frass than either WT males or females (Fig. 3c). In addition, behavioral trials were conducted with either virgin female or virgin male frass vs. a solvent control, and each trial produced statistically identical attraction, with both male and female frass being behaviorally attractive in trap assays (Supplemental 6C). In summary, the data show that frass is a strong attractant across several tested behavioral paradigms for *Drosophila* attraction and aggregation, and that both male and female frass is attractive.

### The Effect of Frass on Feeding Behavior

We conducted three sets of feeding trials, first using food dye to determine the preference of *D. melanogaster* for feeding on substrates infused with frass (Fig. 3a). Regardless of whether red or blue dye was used, flies preferred to feed from solutions containing frass (Fig. 3a; Supplemental Fig. 4). To confirm that flies were feeding in addition to aggregating at the solution, images of the colored dye were taken after the feeding trials were completed (Supplemental Fig. 4). In a second feeding trial, in this case without dye and during 30 min of direct observation with starved flies, the feeding solution containing frass again was significantly preferred over the control solution (Fig. 3b). In addition, we conducted a third set of feeding trials using CAFÉ assays, which compared 5 % sugar water (control) to the same solution with the addition of fecal material (Fig. 3c). In these trials, WT control flies fed more from the treatment containing frass; however, we also observed that ORCO flies preferred to feed from the capillary that contained frass (Fig. 3c), suggesting that while feeding is enhanced by fecal material, that this increase is perhaps not directly influenced by odorant receptors.

### Examination of Frass from Different Species

**Fig. 4:**
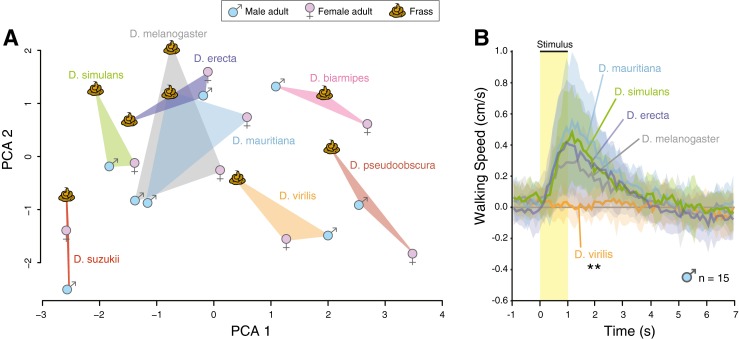
**a** PCA (variance–covariance matrix) of normalized and quantified major peaks within the GC-MS profiles for 8 species of *Drosophila* flies, including adult male, adult female, and adult frass collections. Several species differ significantly from each other (one-way ANOSIM; Bray–Curtis distance; *R* = 0.78; *P* < 0.001), with the melanogaster clade clustering together without significant differences (*D. simulans*, *D. melanogaster*, *D. erecta,* and *D. mauritiana*; *P* > 0.05). The frass samples collected from *D. suzukii*, *D. biarmipes*, *D. pseudoobscura,* and *D. virilis* were all significantly different from each other, and from the *D. melanogaster* clade (*P* < 0.05). **b** Behavioral trials using *D. melanogaster* adults in the Flywalk that were given the choice between frass collected from several different *Drosophila* species. Flies showed no difference in attraction for closely-related species within the same clade, but were not attracted to the frass from more distant relatives such as *D. virilis*

Having shown that frass from *D. melanogaster* contains a sex-specific combination of CHCs and pheromones, our next interest was determining whether different *Drosophila* species contained notable differences in their frass. To test this we examined eight species of *Drosophila* flies, and compared the male and female adult body washes of each species to their corresponding fecal collections. We examined GC-MS data from 600 s onward, which included a total of 69 distinct compounds across the 8 fly species, and the data were normalized to the total amount of peak area in each total ion chromatogram (TIC). Data were log transformed to ensure normality, which was checked by the Shapiro-Wilk test. We used open-source XCMS implemented into the statistical program R to align the raw total ion traces (Smith et al. 2006), which we then used for the PCA, with PCA1 explaining 28 % and PCA2 explaining 16 % of the total variance. In the case of the *melanogaster* clade, all species that we examined produced remarkably similar chemical profiles, not just in the adult body washes, but also in their frass (Fig. 4a; Supplemental 1, 2). While the *melanogaster* relatives (*D. erecta, D. mauritiana, D. simulans*) all produced similar levels of ML, MM, and MP in their frass to that of *D. melanogaster*, there were small differences regarding both cVA content as well as other specific CHCs.

When our analyses was expanded to include more distant relatives of the family Drosophilidae, we were able to demonstrate species-specific differences in fecal deposits (Fig. 4a) in addition to the differences that were observed between adult males and adult females of each species (Fig. 4a; Supplemental 1, 2). Thus, frass appears to provide a chemical signature for each species, and provides species-specific markers to identify as well as leave behind information about the flies that were previously present. In general, the frass that was generated appeared to mirror the adult CHC and pheromone profile. While all examined species and their frass contain pheromone components such as ML, MM, and MP, many species and their corresponding frass appears to be deficient in cVA, further confirming that this compound and other male-produced compounds may be more indicative of species differences than other behaviorally relevant odors. For example, we were able only to identify a minuscule amount of cVA that was generated by *D. suzukii* or *D. virilis*, which had been suggested previously (Dekker et al. 2015), but other species such as *D. biarmipes* appeared to contain larger amounts of this pheromone component in adult male male body washes as well as in collected male frass.

### Attraction of Frass from Different Species

To test for behavioral differences between the frass collected from different *Drosophila* species, we again utilized the Flywalk. Here we tested the response of *D. melanogaster* adult males towards odor pulses from the frass collected from several different species. While *D. melanogaster* adults were equally attracted to 45 mg of frass from closely-related species (*D. melanogaster*, *D. mauritiana, D. simulans, and D. erecta*), they were significantly less attracted to the odor pulses from more distantly related fly species such as the fecal collections from *D. virilis* (Fig. 4b).

## Discussion

In this study, we showed that *Drosophila* frass is behaviorally attractive, and that it provides chemical cues for aggregation in *Drosophila*. Our data also demonstrate that this attraction is predominantly due to the presence of pheromone compounds within the fecal droplets, specifically, the ligands that activate Or47b, Or88a, and Or67d (ML, MM, MP, and cVA, respectively). Moreover, the importance of MM, ML, and MP and their role in aggregation and courtship already has been demonstrated (Dweck et al. 2015). Recent work by Lin et al. (2016) has suggested that several fatty acids (i.e., myristic acid, palmitoleic acid, and palmitic acid) also strongly activate Or47b, and our analyses has shown that these compounds are also all found in high abundance in the frass. It also has been previously established that 7-T and 9-T inhibit mating between species and contribute to aggregation (Fan et al. 2013), and our current study confirmed that these CHC compounds were found in high abundance within the fecal droplets as well. Numerous studies have shown that cVA has roles in aggregation, in mating deterrence, in male-male aggression, and that this compound is passed from males to females as an anti-aphrodisiac during mating (Auer and Benton 2016). Given all this information, our data suggest that frass also could achieve these same behavioral outcomes through the activation of the same neuronal circuits, due to the presence of the before mentioned chemistry (ML, MM, MP, and cVA, as well as their corresponding acids), and thus that frass is to a great extent a general aggregation signal that is composed of robust gustatory and olfactory cues. However, future work is necessary to examine the importance of frass in other *Drosophila* behaviors beyond attraction, such as mate recognition, courtship, male-male aggression, and oviposition.

In subsequent experiments we also generated evidence that the presence of frass increases feeding behavior. Given that this increase in feeding appears to not be mediated by olfactory receptors, as demonstrated by the use of Orco mutants (Fig. 3c), future studies will target the possible role of gustatory (Gr), as well as ionotropic (Ir) and PPK receptors. Since 7-T is detected by gustatory neurons expressing Gr32a (Wang et al. 2011), this receptor might be a candidate in mediating the increased feeding. It also is worth noting that while the contents of *Drosophila* frass have not yet been analyzed specifically for microorganisms, it is likely that this fecal material contains both yeast and bacteria in addition to the described pheromone components. It recently has been shown that specific Grs and Irs are responsible for the increased feeding and mating receptivity afforded by the presence of yeast (Gorter et al. 2016). Therefore, the increased feeding on solutions containing frass is most likely at least partially linked to these same taste receptors, although more work is needed to test this hypothesis, and to further examine the presence of potential microorganisms in *Drosophila* frass.

The frass collected from each sex and each species of fly appears to match the odor profile of the adult that produced it (Fig. 4a). This similarity between adult and frass chemistry is not surprising given that the alimentary canal consists of a cuticular material similar to that which forms the outer epi- and exocuticle. It is thus reasonable that frass content positively correlates to the exterior pheromone and CHC profile of the adult fly (Fig. 4a). The data reported here support the current literature that *Drosophila* can discriminate between species-related chemical differences among adults, but our data go one step further and also support the notion that *Drosophila* can discriminate between the frass or fecal deposits left behind by distantly related species at a food source (Fig. 4a, b). While it has not been shown previously that frass from *Drosophila* contains behaviorally relevant chemical stimuli, this has been demonstrated repeatedly for other insect orders, including Coleoptera and Blattodea (Symonds and Gitau-Clarke 2016; Wada-Katsumata et al. 2015). In research with other insects, frass has also been shown to provide a substrate that can be used to identify novel pheromone components from several agricultural and economic pests, such as the boll weevil and the many destructive species of pine beetle (Bellas et al. 1969; Hall et al. 2002; Symonds and Gitau-Clarke 2016; Tumlinson et al. 1969).

While previous work has identified the presence of pheromones as part of the fecal signature in these insects, it has not been shown that *Drosophila* frass also contains sex-specific and species-specific markers. Therefore, our current investigation of frass chemistry provides several avenues for future application, such as the identification of novel pheromone components from additional insect species, especially in cases where the induction of calling behaviors or where the release of pheromones is difficult to stimulate in the laboratory. Examination of *Drosophila* frass also provides novel approaches to the studies of economically important species within this genus, such as *D. suzukii*, where the loss of cVA might have been replaced by another behaviorally relevant male-generated pheromone component that could be more easily identified from fecal studies. It also is likely that certain chemical components of *D. suzukii* frass could provide species-specific attraction and aggregation cues that in turn may benefit current IPM strategies.

While frass from otherwise healthy adults is behaviorally attractive, it is not yet determined whether diet or other external influences can modify the chemical signature of feces. It would be interesting to address whether the chemistry of frass changes in regard to food resources, such as in *Drosophila* reared upon different food substrates (e.g., food deficient in amino acids or sugars) or by rearing the flies upon the same fruit at different stages of decay. Moreover, it would be interesting to ascertain whether the frass itself changes after exposure to or ingestion of different healthy or pathogenic microbes that have been incorporated into the diet, such as different yeast or bacteria strains. It is possible that frass can provide a signature or snapshot of individual insect health, or perhaps insect population health, especially as it relates to mid- and hindgut metabolism (Kuhns et al. 2012; Newell and Douglas 2014). Additional work is also required to ascertain whether the frass itself affects the substrate that it is deposited onto, namely the fruit or food resource utilized by each *Drosophila* species. While it is clear that frass contains pheromone components, and that frass is involved in the attraction or recruitment of other *Drosophila* to a food source, it still is open for debate whether the frass itself is an active substance that plays any role in breaking down food resources, such as through the utilization of gut microbes, including yeasts or bacteria, or through the use of enzymatic and digestive substances that are potentially deposited along with or within the fecal spots. In the present study, we showed that flies deposit frass in a rather random, but often non-overlapping distribution across the entire exposed surface area of potential food substrates (Fig. 1a). Therefore frass may aid in the decay or fermentation of nutrient resources through the recruitment or deposition of microorganisms. It has already been demonstrated that ingested microbes such as yeast spores can survive the digestive tract of *Drosophila* (Coluccio et al. 2008; Erkosar and Leulier 2014). Thus, it is likely that different species of *Drosophila* produce frass that contains different strains of microorganisms that could in turn be distributed through fecal spots to assist or accelerate the breakdown of species-specific food resources (e.g., cacti, mushrooms, or fruit) (Wong et al. 2013, 2014). This scenario would potentially benefit both the fly and the microorganisms that they in turn vector to each new host plant.

It is clear from the present study that frass contains relevant chemical information for each *Drosophila* species and that fecal deposits appear to play a role in both feeding and aggregation. However, it is not yet clear whether frass plays any additional roles in aspects of courtship, or whether frass affects oviposition decisions, such as site selection. It has been demonstrated that some species of flies such as Tephritids leave oviposition marks that ward off other females (Arrendondo and Diaz-Fleischer 2006). Thus, it is possible that some species of *Drosophila* might utilize similar fecal deposits to mark fruit after oviposition, especially in cases when eggs are either laid singly or where they are laid in tight clusters. A recent study has examined sperm plugs containing cVA that are deposited by mated *Drosophila* females that enhance oviposition (Dumenil et al. 2016). Potentially, we could have overlooked sperm plugs when collecting mated female feces for examination. However, as feces from virgin females and virgin males were both significantly attractive to adult flies (Supplementary Fig. 6C), we can conclude that additional cues besides cVA are involved in fly attraction towards frass. Nevertheless, specific studies examining the effects of frass on oviposition also are still required, and future studies will need to separate the contributions of cVA from the other pheromone cues found in frass. Currently, one of the more economically important *Drosophila* species, *D. suzukii*, would be a prime candidate for a more extensive study of frass in regard to attraction, avoidance or oviposition, as any attractive or deterrent chemistry from frass may aid in IPM strategies towards the control of this pest insect. While we were able to show the presence of cVA in *D. suzukii* for both adult extractions and within male frass, albeit greatly reduced compared to *D. melanogaster*, it is possible that another male-produced compound is still passed from males to females during copulation in this species, and thus frass material may provide an avenue for the identification of such novel chemistry. In summary, as growing evidence continues to support an intimate association between *Drosophila* and distinct microorganisms, it is clear from our study that additional research should be conducted to examine *Drosophila* frass and its role in the chemical ecology of this genus of fly.

## Electronic supplementary material

ESM 1 Supplemental Four species of *Drosophila* within the melanogaster clade were examined via GC-MS. Adult male and female body washes for each species are shown (pink, blue), as well as the frass chemical profile (below). Highlighted are the known pheromone compounds, methyl laurate (ML), methyl myristate (MM), methyl palmitate (MP), and 11-*cis*-vaccenyl acetate (cVA). Few differences between these closely related species are noted, as shown in the PCA analysis of all GC-MS data (Fig. 4A). (PDF 1379 kb)

ESM 2 Supplemental Four distantly related species of *Drosophila* were examined through GC-MS. Adult male and female body washes for each species are shown (pink, blue), as well as the frass chemical profile (below). Highlighted are the known pheromone compounds, methyl laurate (ML), methyl myristate (MM), methyl palmitate (MP), and 11-*cis*-vaccenyl acetate (cVA). Many differences are noticeable between these more distantly related species, as shown in the PCA analysis of all GC-MS data (Fig. 4A). (PDF 1354 kb)

ESM 3 Supplemental Frass was collected from the sides of 1-wk.-old vials by scraping with a round-ended micro spatula. Collection was made from the upper zone of the vial for *Drosophila melanogaster*, avoiding the other distinct lower zones that contained larvae and pupae. Fecal collections were then added to a solvent for use in subsequent GC-MS and behavioral analyses. (PDF 26848 kb)

ESM 4 Supplemental Feeding assays using dye were examined using images of the flies to document the presence of red or blue dye within the abdomen, examples are shown to confirm that flies fed upon the solutions containing frass. (PDF 139542 kb)

ESM 5 Supplemental 5 Schematics for each behavioral assay, including (A) trap assays (B) feeding arenas (C) CAFÉ assays, and (D) the Flywalk. See methods for additional information and references. (PDF 1219 kb)

ESM 6 Supplemental 6 (A) Female and male frass collections from 7-d-old virgins. (B) Table corresponding to the identified chemistry from female and male frass. (C) Trap assay data showing that frass collected from both males and females are significantly more attractive than the solvent control (*P* < 0.05). (PDF 674 kb)
