# Supplementary figures and images for: Adult Frass Provides a Pheromone Signature for Drosophila Feeding and Aggregation
Source: J Chem Ecol. 2016 Aug 18;42(8):739–47. doi: 10.1007/s10886-016-0737-4 (PMC5045843; doi:10.1007/s10886-016-0737-4)

Supplemental Figure 1.

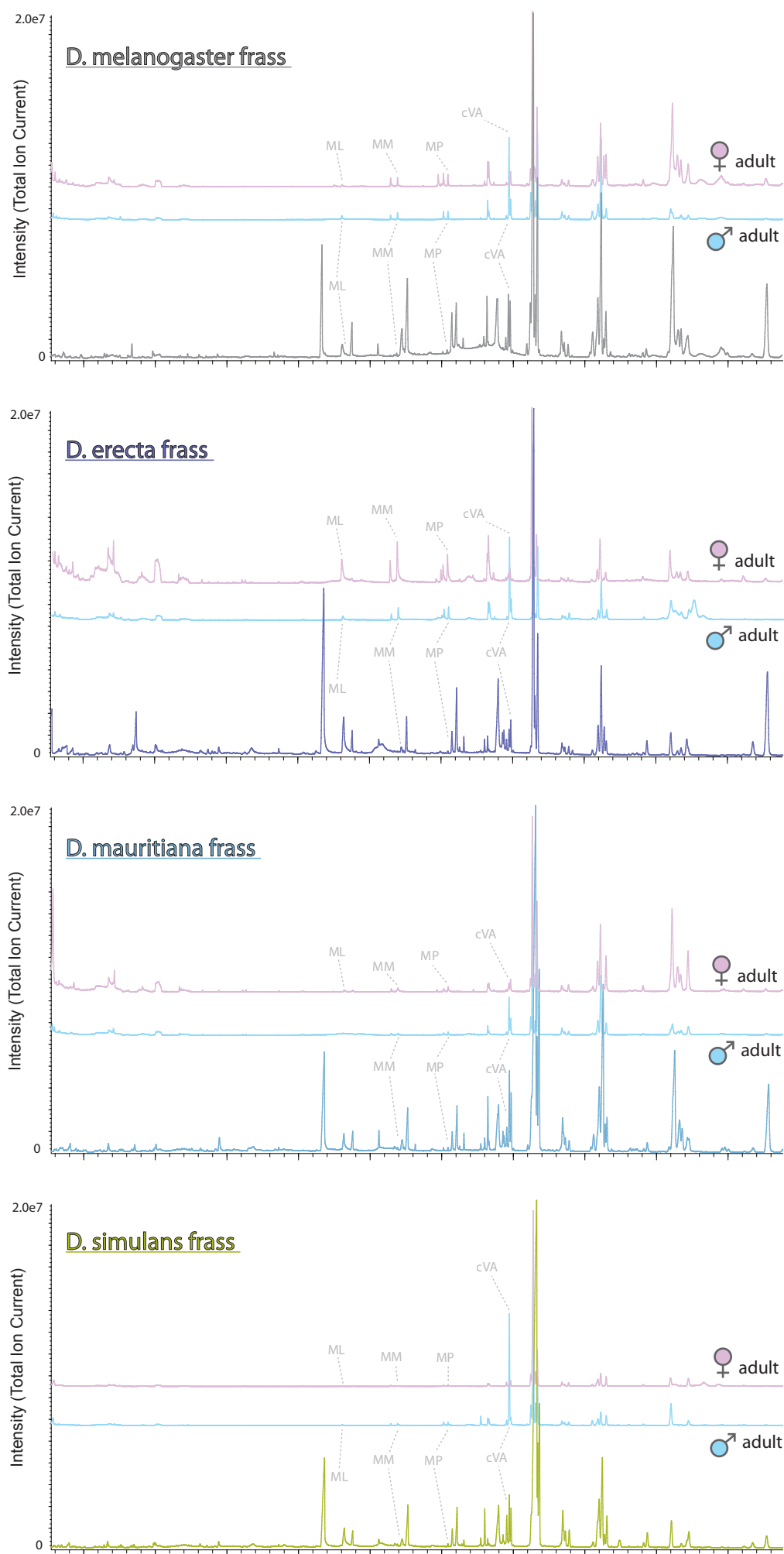

Supplement: Supplementary file 1 — Four species of Drosophila within the melanogaster clade were examined via GC-MS. Adult male and female body washes for each species are shown (pink, blue), as well as the frass chemical profile (below). Highlighted are the known pheromone compounds, methyl laurate (ML), methyl myristate (MM), methyl palmitate (MP), and 11-cis-vaccenyl acetate (cVA). Few differences between these closely related species are noted, as shown in the PCA analysis of all GC-MS data (Fig. 4A). (PDF 1379 kb) [file 10886_2016_737_MOESM1_ESM.pdf]

Supplemental Figure 2.

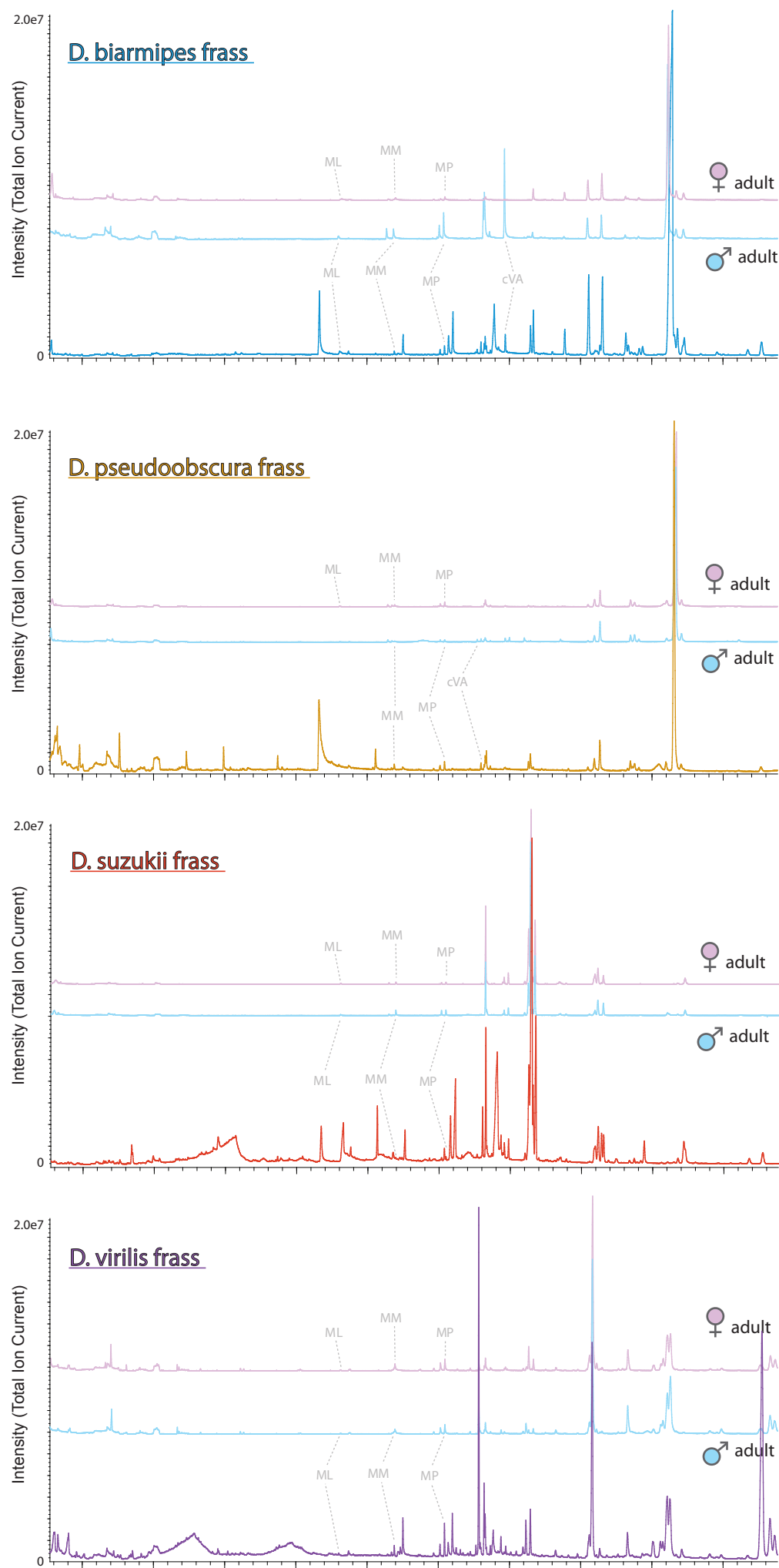

Supplement: Supplementary file 2 — Four distantly related species of Drosophila were examined through GC-MS. Adult male and female body washes for each species are shown (pink, blue), as well as the frass chemical profile (below). Highlighted are the known pheromone compounds, methyl laurate (ML), methyl myristate (MM), methyl palmitate (MP), and 11-cis-vaccenyl acetate (cVA). Many differences are noticeable between these more distantly related species, as shown in the PCA analysis of all GC-MS data (Fig. 4A). (PDF 1354 kb) [file 10886_2016_737_MOESM2_ESM.pdf]

Supplemental Figure 3.

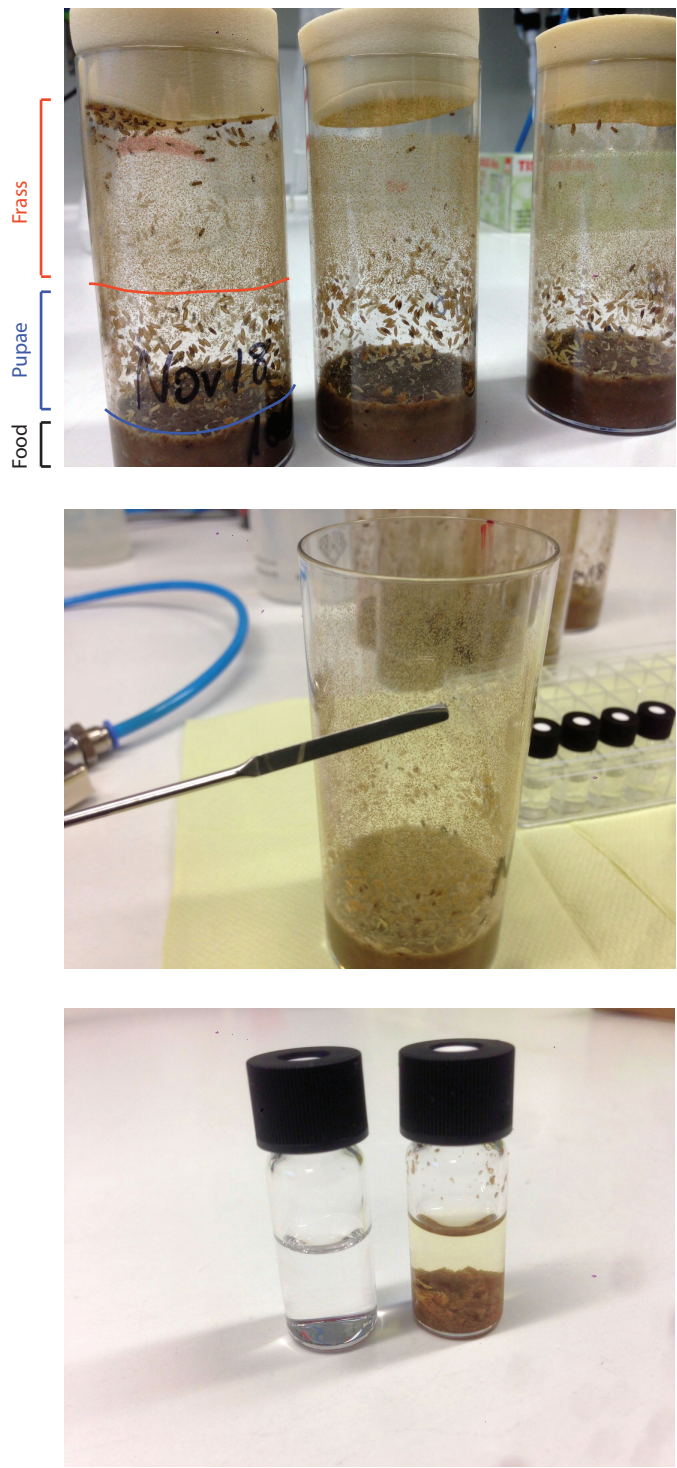

Supplement: Supplementary file 3 — Frass was collected from the sides of 1-wk.-old vials by scraping with a round-ended micro spatula. Collection was made from the upper zone of the vial for Drosophila melanogaster, avoiding the other distinct lower zones that contained larvae and pupae. Fecal collections were then added to a solvent for use in subsequent GC-MS and behavioral analyses. (PDF 26848 kb) [file 10886_2016_737_MOESM3_ESM.pdf]

Supplemental Figure 5.

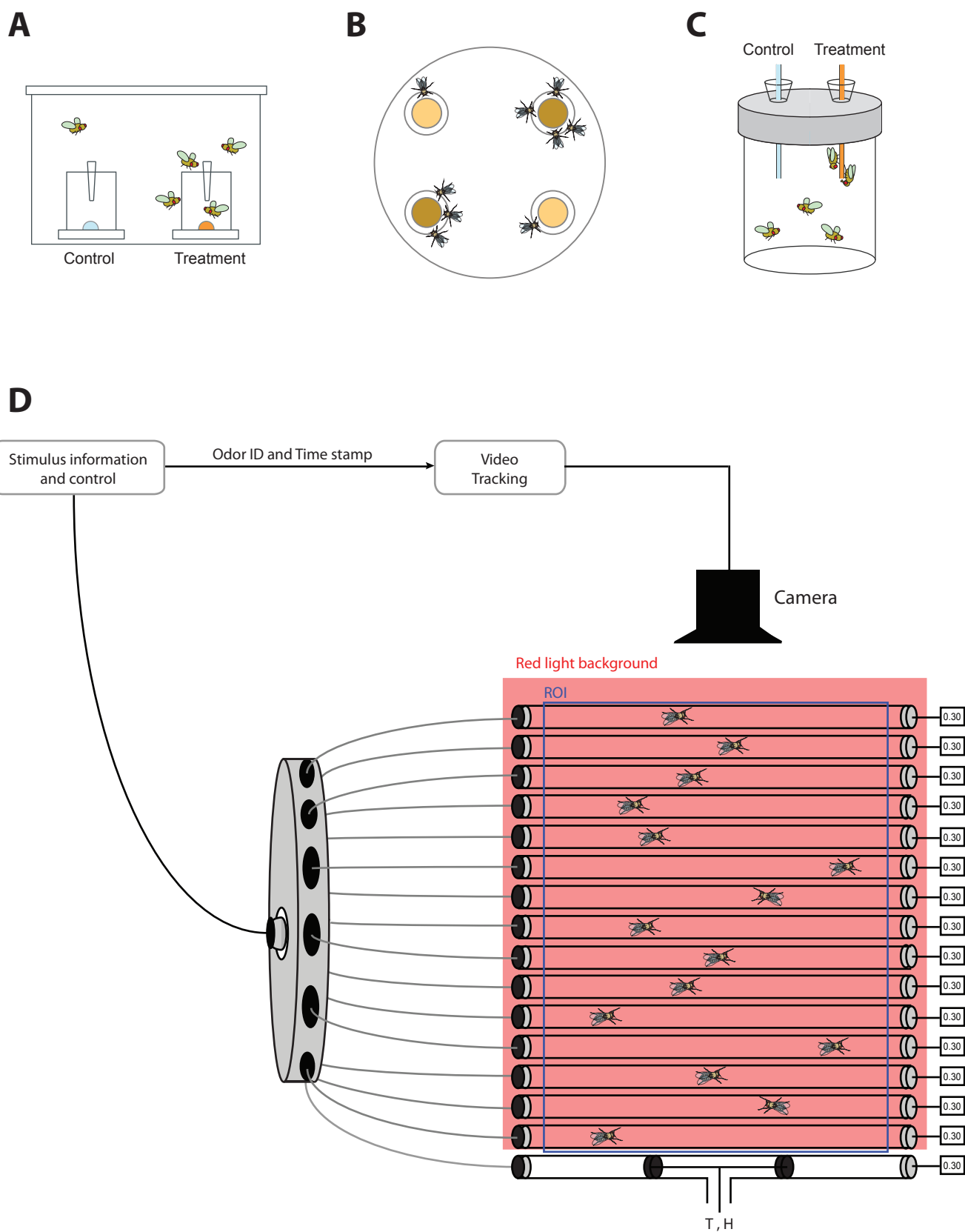

Supplement: Supplementary file 5 — Schematics for each behavioral assay, including (A) trap assays (B) feeding arenas (C) CAFÉ assays, and (D) the Flywalk. See methods for additional information and references. (PDF 1219 kb) [file 10886_2016_737_MOESM5_ESM.pdf]
