# Supplementary material for: Adult Frass Provides a Pheromone Signature for Drosophila Feeding and Aggregation
Source: J Chem Ecol. 2016 Aug 18;42(8):739–47. doi: 10.1007/s10886-016-0737-4 (PMC5045843; doi:10.1007/s10886-016-0737-4)

Supplemental Figure 6.

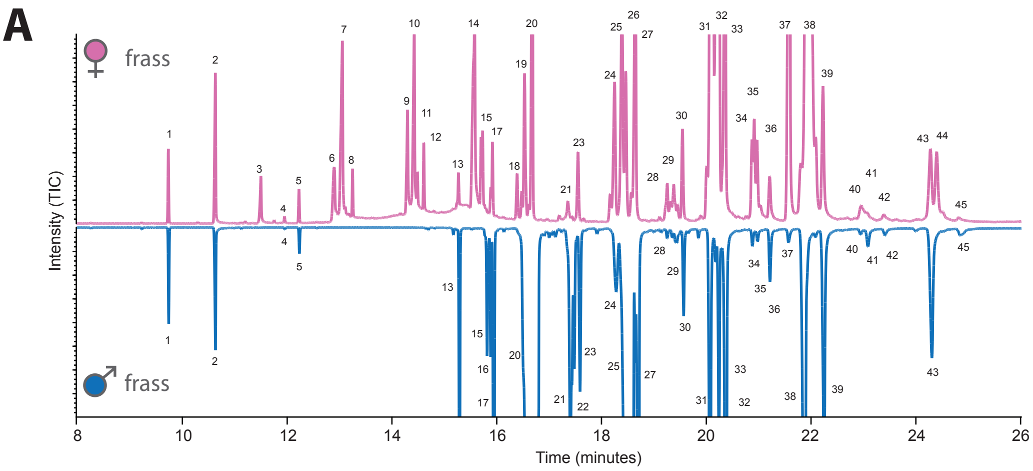

B

Peak No.	Kovats Index	Compound Name	Male	Female
1	1329	Bromodecane (internal standard)	+	+
2	1365	Methylparaben	+	+
3	1498	Lauric Acid	-	+
4	1527	Ethyl Laurate	+	+
5	1545	N-Pentyl-decanamide	+	+
6	1723	Myristoleic Acid	-	+
7	1724	Myristic Acid	-	+
8	1726	Ethyl Myristate	-	+
9	1925	Methyl Palmitoleate (Palmitoleic Acid)	-	+
10	1926	Palmitic Acid	-	+
11	1928	E-9-Hexadecenoate	-	+
12	1931	Ethyl Palmitate	-	+
13	2165	Heneicosane	+	+
14	2188	Linoleic Acid	-	+
15	2190	Ethyl Oleate	+	+
16	2194	(Z)-11-Vaccenyl Acetate (cVA)	+	-
17	2195	Heneicosane	+	+
18	2272	7(Z),11(Z)-Heptacosadiene	-	+
19	2274	(Z)-9-Tricosene	-	+
20	2275	Heneicosane	+	+
21	2279	Cyclotetracosane	+	+
22	2281	(Z)-7-Tricosene	+	-
23	2284	Tetracosane	+	+
24	2481	(Z)-14-Tricosenyl Formate	+	+
25	2487	(Z)-12-Pentacosene	+	+
26	2493	(Z)-12-Pentacosene	-	+
27	2498	Octacosane	+	+
28	2541	11-Hexacosyne	+	+
29	2543	9-Hexacosene	+	+
30	2547	Hexacosane	+	+
31	2665	7(Z),11(Z)-Heptacosadiene	+	+
32	2669	1-Heptacosanol	+	+
33	2772	Heptacosane	+	+
34	2791	Unknown	+	+
35	2799	Unknown	+	+
36	2804	Tetratetracontane	+	+
37	2821	Squalene	+	+
38	2832	7(Z),11(Z)-Nonacosadiene	+	+
39	2837	Hexatriacontane	+	+
40	2901	1,30-Triacontanediol	+	+
41	2902	Unknown	+	+
42	2912	Unknown	+	+
43	2934	Hentriacontane	+	+
44	2936	Tetracontane-1,40-diol	+	+
45	2951	Tetratriacontane	+	+

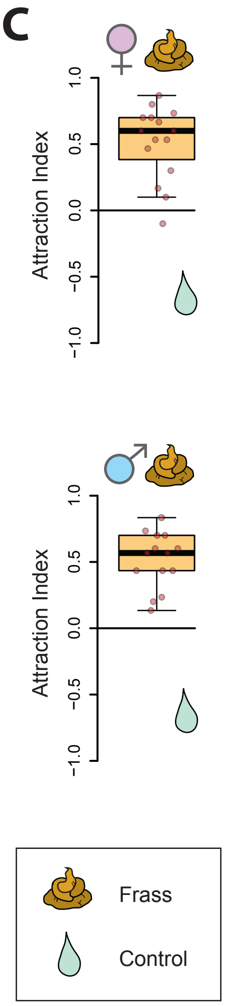

Supplement: Supplementary file 6 — (A) Female and male frass collections from 7-d-old virgins. (B) Table corresponding to the identified chemistry from female and male frass. (C) Trap assay data showing that frass collected from both males and females are significantly more attractive than the solvent control (P < 0.05). (PDF 674 kb) [file 10886_2016_737_MOESM6_ESM.pdf]
